# Comparative efficacy of three pediculicides to treat head lice infestation in primary school girls: a randomised controlled assessor blind trial in rural Iran

**DOI:** 10.1186/s12895-019-0093-5

**Published:** 2019-09-12

**Authors:** Hadi Kalari, Aboozar Soltani, Kourosh Azizi, Hossein Faramarzi, Mohammad Djaefar Moemenbellah-Fard

**Affiliations:** 1 Department of Medical Entomology and Vector Control, School of Health, Shiraz University of Medical Sciences, Shiraz, Iran; 2 Research Center for Health Sciences, Institute of Health, Department of Medical Entomology and Vector Control, School of Health, Shiraz University of Medical Sciences, Shiraz, Iran; 3 Department of Community Medicine, School of Medicine, Shiraz University of Medical Sciences, Shiraz, Iran

**Keywords:** Pediculosis, Topical treatment, Head lice, *Pediculus capitis*, Dimeticone, D-phenothrin, Iran

## Abstract

**Background:**

Head lice infestation (Pediculosis) is one of the most important health challenges particularly in primary school-aged children. It is often present among 6–11-year-old students in various tropical and temperate regions of the world. The aim of this study was to examine epidemiologic indices and comparative analysis of two pyrethroid-based and one non-chemical pediculicide products on head lice treatment of primary school girls in a rural setting of Fars province, south Iran, as part of a randomized controlled assessor blind trial.

**Methods:**

Before treatment, infested students were screened using plastic detection combs to find live head lice. Three independent parallel groups, each with about 25 participants (#77) were eventually twice with a week apart treated with either 1% permethrin, 0.2% parasidose (d-phenothrin) or 4% dimeticone lotion preparations. In each case, a questionnaire form was completed on epidemiologic factors. Data were registered after a fortnight from primary scalp treatment and re-inspection on days 2, 6, 9 and 14. Data analyses were performed using Chi-square test with a *P*-value < 0.05 being taken as statistically significant.

**Results:**

From 3728 inspected students, 87 (2.33%) girls were infested with head lice, *Pediculus humanus capitis* De Geer, 1778. Ten students dropped out pertaining to exclusion criteria. No significant correlation was found between head lice infestation level and hair length, hair style, itching, nationality, age, settlement site and baths; but there was a significant relationship between age and hair style (*P* = 0.027). The efficacy values on each of the above re-inspection days from each of the three treatments were 81, 74, 70 and 63% for permethrin; 83, 92, 100 and 100% for dimeticone; and 96, 88, 96 and 92% for d-phenothrin; respectively. A quartile difference in efficacy of permethrin relative to dimeticone on day 14 represented the scale of head lice resistance to permethrin treatment. There were significant statistical differences in case re-inspection days 9 (*P* = 0.008) and 14 (*P* = 0.003) post treatment. Only two dropout cases, one non-compliant and the other lost before the second-week treatment, from permethrin trial were observed following two applications a week apart.

**Conclusions:**

Dimeticone lotion had the fullest efficacy (100%) among all treatments. This high cure rate was attributed to the low level of infestation and the extent of patients’ involvement. Parasidose swiftly ameliorated the infested cases by the second day since initial treatment. Female third grade students were the most infested cohort.

**Trial registration:**

Current Controlled Trials- IRCT2016041627408N1, Dated: 21-08-2017.

## Background

In most resource-poor settings of the developing world, it is common to perform clinical trials in an area where products are almost freely available to residents. In addition, executive staff at the center for disease control often has to obtain data from these areas to assess the disease burden under surveillance by situation analysis at a given spatiotemporal profile. To control louse infestation, the initial intensive use of various insecticides could have selected for resistant head lice populations. The treatment outcome in a remote region, where use of these products has been negligible, could overtly be different from that where insecticides are extensively applied.

Given the roughly two-weeks-long life of head lice spent on man, considerable biological activity and damage could be incurred on its human host. Pediculosis capitis or infestation with the human head lice, *Pediculus humanus capitis* De Geer, 1778 (Anoplura: Pediculidae), an obligatory ectoparasitic hematophagous insect on man [1], is still a persistent health menace to many socioeconomic groups worldwide. This disease is not self-healing, almost insidious and irritating [2]. It has been reported from many endemic parts of the world including Iran [3, 4], where a plethora of other infectious diseases coincidentally occur [5–7]. Although all age groups are vulnerable, pediculosis is particularly more often observed on primary school-aged children about 3–11 year old [8, 9]. It has a striking impact on the well-being of children and their school attendance since it is infectious. If it is not treated, secondary microbial infection could exacerbate the illness [2], leading to impetigo and dermatitis. Despite increasing knowledge on head lice control, burden of this infestation in communities has remained unbearable.

Previous studies have documented unequivocal evidence on the presence of strains of head lice refractive to several pediculicides, insecticide-based shampoos against lice [10–13]. For instance, permethrin has previously been, or is still, the main source of head lice therapy in the developed world. It is a powerful pyrethroid insecticide derived from the *Chrysanthemum* flowers. Permethrin, a contact insecticidal product, is available as over-the-counter (OTC) product for pediculosis treatment. It may still be safe to apply intermittently in certain specific settings. Application of permethrin and lindane, a non-aromatic neurotoxic organochlorine insecticide, have produced comparable results in an earlier study [3], but permethrin was generally found to be more effective in a systematic review [14], while use of lindane is no longer recommended. The use of lindane has been prohibited since 2007 by the European Union [15].

Detection of insecticide resistance is not a trivial task. The persistence of living *P. h. capitis* after application of a pediculicide could have several causes, including: lack of compliance of the participant to the treatment protocol; wrong treatment (misuse or under-dose); no residual killing or ovicidal effects of the product, resulting in self-re-infestation; re-infestation (re-acquisition of head lice post-treatment); and authentic non-susceptibility of lice to the pediculicide [15].

Efficacy is expressed as the cure or lice-free rate, while the latter means the proportion of patients on whom no living head lice (nymphs or adults) are discovered by a specific method at a specified time point [16]. There is a preliminary report that even in the presence of knock-down resistance (kdr-like) gene, treatment of head lice with permethrin was achieved in 93% of German children whose lice had the kdr-like gene [13]. This efficacy claimed by the producers is not, however, substantiated by valid scientific reports [17]. Another pyrethroid insecticide is d-phenothrin (parasidose or Sumithrin). This is similar to permethrin which is a type 1 pyrethrin analog that lacks an α-cyano substituent. The pediculicide, d-phenothrin, has a low level of toxicity. Resistance to permethrin and d-phenothrin has been recorded in a few European countries [18].

Dimeticone lotion is a silicon-based organic polymer compound known as polydimethylsiloxane. This is a physical rather than chemical compound with no conventional insecticide activity. It is a hydrophobic, colorless and odorless fluid applied by covering the scalp and full length of the hair. Dimeticone consists of a long chain linear silicone (dimeticone) of 100 K centistokes (cSt) viscosity in a volatile solvent of silicone base, decamethylcyclopentasiloxane (cyclometicone D5), with low (2.4 cSt) viscosity and low surface tension (0.018 N/meter). This mixture is allowed to dry up gradually by evaporation of the latter [10]. The silicone chain then impedes the spiracles and tracheal system of head lice preventing water excretion, and this inability to expel excess water causes osmotic pressure on somatic cells leading to their eventual turgidity and rupture, culminating in lice death [19].

Both dimeticone lotion and d-phenothrin liquid share similar physical form and dosage protocols (overnight or 12 h application) which is why they were selected in this research. Since patterns of resistance is determined by the local geographical conditions, and the fact that concrete evidence from randomised controlled trials for any form of treatment is limited in our region, this study was thus undertaken. Thus, the main aim of this investigation was to study epidemiologic characteristics and comparative analysis of three topical pediculicide-based shampoos on head lice treatment of primary school girls in a rural setting of Fars province, south Iran, as part of a randomised controlled assessor blind trial.

## Patients and methods

### Study area

This investigation was conducted in the county of Kavar (52°43′41‶E, 29°11′32‶N at an altitude of about 1386 m above sea level) almost 45 km to the southeast of the capital city of Fars province, Shiraz, Iran. The mean annual ambient temperature was 22 °C and its mean annual ambient relative humidity was 65%. Its population was 83,883 in 2016. The study period was from Dec 2016 to Jul 2017.

### Participants

**Fig. 1:**
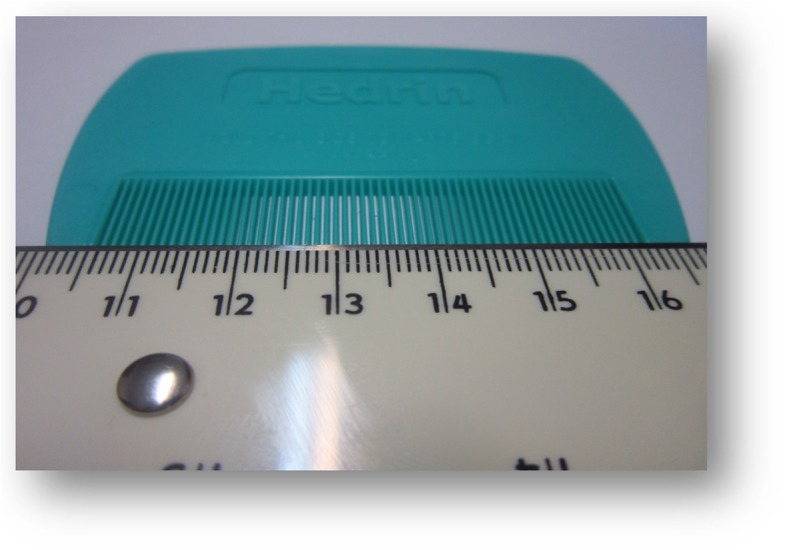
The curved (lower, facing scalp) side of the plastic detection comb (PDC)

Since the recruitment of all primary school students was very laborious to achieve, a representative sample of 21 female schools was selected based on the relatively high incidence of pediculosis in previous years and all their registered female students were screened and then searched for the presence of live *P. h. capitis* first by visual search of the hair and scalp and then by dry-combing or applying standard plastic detection combs (PDC) (Thornton and Ross Ltd., Huddersfield, UK) (Fig. 1). Visual inspection of viable head lice (nymphs and adults) was done with an X10 magnifying lens. Any head lice found by dry-combing was left in the hair to avoid bias. Combing was terminated as soon as live head lice were observed.

All participants were delivered handouts to take home so that any likely lice-infested family members could participate in free treatment, but the latter were excluded from final analyses. All relevant variables including the infestation and itching levels, hair style and length, nationality, age, settlement site and baths were recorded in an epidemiologic form. A signed informed assent form was also completed for each student in accordance with the principles of the revised Declaration of Helsinki, 2013.

### Design and eligibility

This trial was a randomized controlled assessor blind trial to evaluate the efficacy of 1% permethrin (control) versus 0.2% parasidose (d-phenothrin) or 4% dimeticone in the treatment of head lice. To ensure that this trial remained assessor-blinded, the following measures were adopted. The applicators of the pediculicide treatments were prevented from getting aware of the product type by pouring liquids into blank, unmarked and serially-coded vials (25 ml). Although the products’ physical attributes (odor, fluidity, color, etc.) were different, assessor was further blinded by staff rotation and physical isolation of treatments at the sites. Care givers were requested not to divulge the treatment type to assessor. Case report form (CRF) data collection and assessment were also performed by blinded study staff (not recruited in the treatment) to avoid any product bias. Participants and their guardians were not able to find out which treatment applications they were assigned to. Data analysts were blinded to the identity of each treatment group too.

The exclusion criteria included scalp sensitivity to any one of the administered products, scalp hair and head skin disorders and damage such as burns, lesions, etc., secondary microbial infections, use of hair gels, bleach or oils, use of any drugs or medication, or pediculicide-based shampoos within the previous 4 weeks, favism (glucose 6-phosphate dehydrogenase, G6PD, deficiency) and non-compliance with the protocol. Any one of these may influence the study outcome. The inclusion criteria involved all female primary school children aged 6–13 year harboring one or more head lice using PDC following a standard protocol as set out below.

### Ethics

Ethical approval was granted by Shiraz University of Medical Sciences, Vice-Chancellorship for Research and Technology, Ethics Committee (Code of Ethics: IR.SUMS.REC.1396.24). All procedures performed in this study involving human participants were conducted according to the international guidelines for clinical trials with pediculicides in agreement with the principles of the 1964 Declaration of Helsinki and its later amendments in 2013 or comparable ethical standards at the national and international levels. As participants were all below legal age, their parents or guardians signed a form giving written consent for their participation in treatment and stating that they realized the study objectives as outlined in information brochures. This study was also registered in the Iranian Registry of Clinical Trials (IRCT).

### Sample size and random allocation

A sample size of about 80 infested individuals was calculated to be enough for this trial. Those eligible girls meeting the entry criteria were randomly allocated to receive one of the three designated head lice products by a computer generated list in balanced blocks of seven. Treatment assignment was performed by numbered sealed enveloped batches of seven. Extra duplicates were set aside in case of code breakage by participants. At the start of treatment, patients were treated by the next code available at the investigator’s disposal.

### Interventions

Permethrin 1% crème rinse was originally supplied in 60 ml plastic bottles, parasidose (d-phenothrin) 0.2% in 150 ml bottles and dimeticone 4% lotion in 60 ml bottles. Each of these three products were applied to dry scalp using thoroughly shaken liquid and rapidly rinsing until the hair was fully saturated. The products were administered a few drops at a time, dispersing the liquid all over the hair with plastic gloves-covered fingers. To ensure complete coverage, the hair was combed with a normal wide-toothed comb to spread treatment evenly. The hair was left to dry naturally without the use of hair drier or towel drying. During treatments, permethrin, d-phenothrin, or dimeticone were left to remain in place for 20 min, 8 h, or 8 h, respectively; the hair was then washed off with water and allowed to dry naturally at the end of these periods. These treatments were repeated a week later. Each participant received the same product type as before. They were advised not to engage in any non-compliance activities such as the use of pediculicide-based shampoo at home, wet- or dry-combing, removing lice, etc. during this research trial.

### Outcomes

At first screening and on post-treatment days, an indication of the level of head lice infestation was followed according to the frequency with which *P. h. capitis* was found on hair. Three (heavy, medium and light) infestation levels were recognized as: > 1 louse per first stroke of the PDC comb, only 1 louse per first stroke, and 1 louse only after 5–6 strokes of the comb, respectively [20].

The primary outcome measure was obliteration of infestation after fulfillment of the treatment regimen. Each patient was assessed for the presence of head lice on days 2, 6, 9 and 14 after the initial treatment using the PDC comb on dry hair in the same way in which the first screening was performed at the school. Inspections on days 2, 6, and 9 were restricted to 2–3 strokes of the comb on each section of the hair [21]. This aimed to give snapshot data of the status of infestation, since more frequent combing could be considered as extra intervention, thus confounding the results. There was no limit of PDC combing frequency on day 14. The lice-free rate or cure was thus defined as no lice after the second application of products, on days 9 and 14. Wet combing was practiced on these post-treatment days. Reinfestation was expressed as the transmission of head lice to individuals during the clinical trial. It was arbitrarily taken as when no more than 2 adult lice or third stage nymphs and no first/second stage nymphs were observed during PDC combing, on days 9 and 14.

The secondary outcome measures included the amelioration of pruritus severity, clinical pathology, and reported adverse events. The level of itching was assessed by a visual analog scale as outlined before [22]. Clinical symptoms involved the presence of erythema, eczema, and dermal weal. Adverse events like intolerable irritations included all health-related parameters, which could postpone or result from treatments.

### Statistical analysis

This descriptive analytic study comprised the mean, frequency, and percentage to describe demographic variables in infested participants. Analyses were performed based on both the intention-to-treat (ITT) and the per-protocol (PP) populations. Differences in efficacy rates between different treatments were calculated by the 95% confidence interval, quantified using a normal approximation to the binomial distribution. One-way analysis of variance (ANOVA) was used to compare the numerical response data from patients. The Chi-square test was applied to compare treatment groups. The statistical package for the social sciences (SPSS) version 19 was applied to analyze the data. A *P*-value of less than 0.05 was considered to be statistically significant in all tests.

## Results

**Fig. 2:**
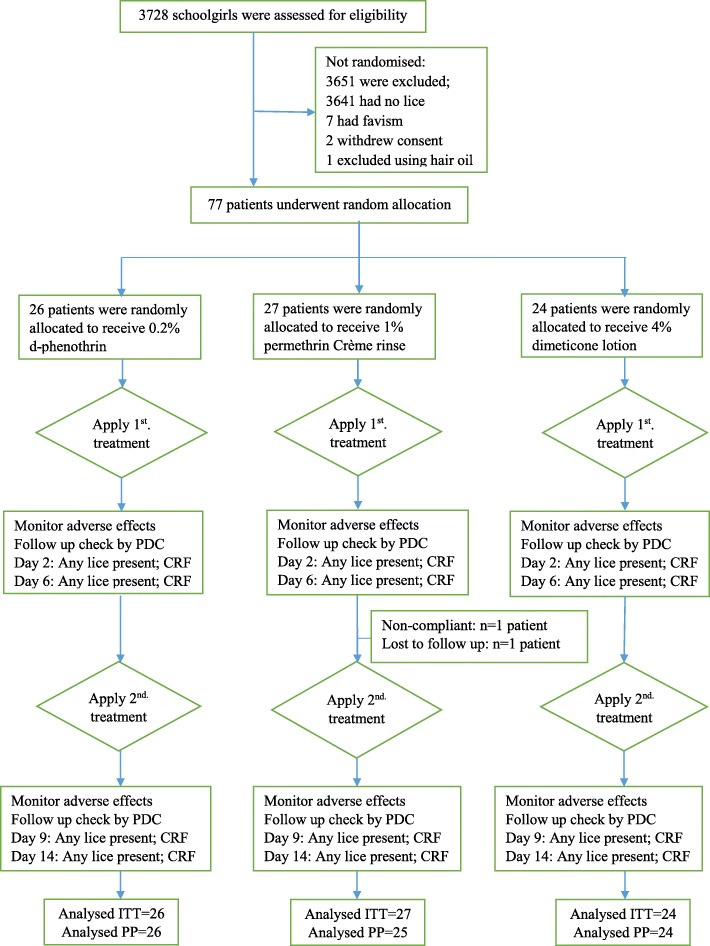
CONSORT flowchart of infested participants’ progress through the study

From an overall population of 3728 female primary school students screened for head lice infestation at Kavar, Fars province, Iran, 87 (2.33%) girls were infested with live head lice. Before the start of trial, 10 dropouts were recorded; seven of them had favism (G6PD deficiency), two withdrew consent and one used hair oil. Therefore, a total of 77 participants entered the treatment trial. Following the second application of permethrin treatment, two further dropouts were noted; one violating the study protocol, non-compliant dropout on day 7 and another one lost to follow-up on day 8 were recorded from permethrin treatment group. The ITT population thus involved 77 infested school children, but 75 participants (PP) fulfilled the study as required by the protocol (Fig. 2).

### Baseline data

Demographic attributes of the participating population at the baseline are presented in Table 1. No significant statistical association was found between the different epidemiological variables and the infestation level (Table 1). The participants had an age-range of 6–13 years, the majority (64%) of whom had light infestation level (i.e. 1 louse/5–6 strokes of PDC comb). The 9-year old students were the most infested (29%) age group. The age-specific infestation level revealed that 7–9-year-old students, in relation to other age groups, were mostly (44%) harboring head lice lightly, whereas the 9–10 year old students’ category constituted slightly less than a quartile (22.7%) of moderate and heavy infestation levels.

**Table 1:** Comparison of demographic characteristics of participants in three treatment groups

	d-Phenothrin 0.2% (#26)	Permethrin 1% (#27)	Dimeticone 4% (#24)	*P*-value
Age in years				
Range	6–13	6–13	6–13	
Mean	1.08	1.32	1	
Median	1	1	1	
	Participants (%)	Participants (%)	Participants (%)	
Hair length				
Above ears	9 (35)	9 (33)	8 (33)	0.751
Ears to shoulders	12 (44)	8 (30)	8 (33)	
Below shoulders	5 (19)	10 (37)	8 (33)	
Infestation				
Light	15 (58)	17 (63)	17 (71)	0.646
Medium	5 (19)	6 (22)	3 (12)	
Heavy	6 (23)	4 (15)	4 (17)	
Itching				
Little	5 (19)	8 (30)	17 (71)	0.129
Moderate	12 (46)	9 (33)	7 (29)	
High	4 (15)	8 (30)	2 (8)	
Very high	5 (19)	2 (7)	3 (24)	
Hairstyle				
Straight	20 (77)	23 (85)	18 (75)	0.470
Curly half	6 (23)	4 (15)	6 (25)	
Frizzy	0 (0)	0 (0)	0 (0)	
Bath				
1/week	20 (77)	25 (93)	17 (71)	0.063
> 2 weeks	6 (23)	2 (7)	7 (29)	

The groups were generally similar with no significant difference in demographic (e.g. hair length, hair style, etc.) features. There was, however, a non-significant association for a larger proportion of children with short (i.e. above shoulders) straight hair compared to the other groups. There was also a non-significant difference between the groups with respect to infestation and itching levels (*P* = 0.08). More than three quarters of all infested participants were found to be in the lower two (i.e. little and moderate) categories of itching grades. The majority of infested students (80%) had straight hair and a bath per week, while the remaining (20%) minority took 2 weeks or more to have a bath.

### Outcomes

The primary outcome analyses included 77 participants who comprised the intention to treat (ITT) population. Three groups of 26, 27 and 24 patients were allocated to receive d-phenothrin 0.2%, permethrin 1% and dimeticone 4%, respectively. Of these 25/26 (96%) and 24/26 (92%) in the d-phenothrin 0.2% group were free from head lice on assessment days 9 and 14 after the second treatment on the day 7 (Table 2). In contrast, the corresponding percentages in the permethrin group were 70 and 63%, whereas in the dimeticone group these were 100%. There were significant statistical differences between the three protocol outcomes on these post-treatment days (*P* = 0.003).

**Table 2:** The therapeutic data in terms of numbers and percentages (%) on different days (D) of treatments as indicated

Treatments	Infested (%)	D2	D6	D9	D14
d-Phenothrin 0.2%	26 (34)	25 (96)	23 (88)	25 (96)	24 (92)
Permethrin 1%	27 (35)	22 (81)	20 (74)	19 (70)	17 (63)
Dimeticone 4%	24 (31)	20 (83)	22 (92)	24 (100)	24 (100)

Following two applications of each treatment regimen a week apart, the first to third rank in terms of efficacy on day 14 was attributed to dimeticone 4%, d-phenothrin 0.2% and permethrin 1%, respectively. In other words, failure in treatment on day 14 after the first administration was 0 % for dimeticone, 8% for d-phenothrin and 37% for permethrin. There was thus a wide gap in efficacy between dimeticone and permethrin treatments.

Preliminary assessment revealed that after the first treatments on days 2 and 6, no significant statistical differences were found between any two protocols with probabilities of 0.29 and 0.26, respectively. After the second intervention, there were significant statistical differences between different treatment protocols on days 9 (*P* = 0.008) and 14 (*P* = 0.003). On day 9, the efficacy of dimeticone compared with d-phenothrin was not in sharp disparity (*P* = 0.337). In contrast, the efficacy of permethrin compared with either dimeticone (*P* = 0.011) or d-phenothrin (*P* = 0.038) was statistically different on day 9 (Table 3). The same trends as above were also observed on day 14. The efficacy of d-phenothrin in comparison to dimeticone was not sharply different on the final day of trial, i.e. day14 (*P* = 0.17). There was, however, considerable discrepancy between the efficacy of permethrin and that of either dimeticone (*P* = 0.003) or d-phenothrin (*P* = 0.027), a fortnight after initial intervention. No serious adverse events were noticed during these trials.

**Table 3:** Comparative percentage probabilities (*P*-values) of treatments on different assessment days

Products/ Days	D2	D6	D9	D14
Permethrin/ Dimeticone	0.950	0.142	0.011	0.003
Permethrin/ d-Phenothrin	0.149	0.248	0.038	0.027
Dimeticone/ d-Phenothrin	0.135	0.709	0.337	0.170

## Discussion

The prevalence rate of head lice infestation in the present study was in accordance with that obtained in an earlier study from another remote region [3]. No considerable differences were noticeable in baseline parameters between the three treatment groups. The 9-year old students were the most infested (29%) age group, since they were also the most abundant participating school aged children. This is a crucial period during which behavior changes could rapidly occur. Before this time, school girls are usually nurtured by their mothers or their elder siblings. After it, they are gradually left to become independent. These and other causes could partly explain why this age group is vulnerable and mostly found to be infested with head lice in various studies [23, 24]. This study was conducted after increasing local complaints from parents on their children’s health at school. Some of them used home remedies such as application of vinegar, ash, Mayonnaise sauce, etc. on hair to get rid of head lice, but they were advised not to engage in such baseless and bizarre activities. Pediculosis capitis is really neglected both at the national and individual’s family level in Iran, where multiple other infectious diseases could simultaneously occur in high risk groups of people [25–27].

Nowadays, resistance-prone insecticide-based products for the treatment of pediculosis are gradually going to be replaced by alternative new preparations with a physical mode of action. These silicone-based products are both safe and clinically effective [10, 20–22, 28, 29]. It is found in this study that dimeticone 4% is more efficacious in curing head lice infestations than either 1% permethrin or 0.2% d-phenothrin solutions using two interventions a week apart. This finding is in accordance with previous reports [21]. It is thus obvious from this randomized controlled trial that dimeticone efficacy clearly outweighed that of permethrin. A silicone-based physically acting preparation was superior in overall activity to an insecticide-based product affected by pediculicide resistance. The reason why permethrin 1% is selected in this study as the comparator product instead of any other alternative preparations, lies in the fact that this was readily available as an OTC product in the market. It was also routinely used by CDC personnel to curb head lice infestations in different parts of Iran.

Parasidose or d-phenothrin is not commonly present in drug stores, but it was recommended to be compared with other new products such as dimeticone. In addition, the level of insensitivity to permethrin (37%) was almost 5-fold that of d-phenothrin (8%) at the end of this treatment trial.

It is generally envisaged that long treatment period is more effective than short term applications when administering head lice products. This is revealed in a small scale study which demonstrated that an overnight application of 4% dimeticone lotion was more efficacious than one of only 20 min [10]. In contrast, a liquid gel formulation of this compound not only diminished its treatment time to 15 min, but also a single intervention seemed to be sufficient in obliterating head lice infestations [30].

Wet combing on post-treatment days 9 and 14 was very efficient to assess and diagnose active lice infestation. Unlike previous reports [31], efficacy was evaluated both 1 day and 7 days after the last treatment, as more than 90% of girls appeared louse-free 1–2 days after a second intervention; but it does not follow that further checks are not indispensable as some viable lice may remain.

The strength of the present study was that participants and their guardians adhered to the strict directions exerted on them during each treatment practice. The opportunity of comparing three different anti-head lice products was another item of strength which hardly occurred in previous publications in this field of health sciences in this country. During this trial, it was assumed that the treatments were consistently applied between schools and that there were no relevant differences between the school populations. Under such circumstances, we ignored any potential clustering within schools. Although clustering with a multilevel model with a random intercept for schools would have been desirable to investigate, but the means of implementing this item was not included at the start of trial. One of the limitations in the current trial was that misuse and overexposure of permethrin during earlier periods could have led to its unacceptable low efficacy.

This report validates previous findings [10, 19–22] on the remarkable efficacy of dimeticone in head lice treatment. It is thus logical to refrain from using insecticide-based products, particularly in an area where insensitivity to their application has been recorded. The application of a product with a physical mode of action rather than chemically-based preparations to control head lice is highly recommended in the future. Furthermore, head lice control in a population depends on coordinated home-based attempts to cure all patients. Isolated treatment of a small group of school aged children not only does not lead to amelioration and relief from head lice, but may also risk rapid reemergence of infestations among the same population of students. This would have enormous repercussions on community health in the long term.

## Conclusions

This randomized controlled trial has demonstrated that there was no significant difference between the efficacy of 4% dimeticone lotion and d-phenothrin product. It is therefore concluded that the superior efficacy of dimeticone over permethrin conduces to the shifting use of the former instead of the latter.

## Data Availability

The data are available from the corresponding author on reasonable request.

## Abbreviations

°C: Degree centigrade, the unit of temperature
ANOVA: Analysis of variance, a statistical test
CRF: Case report form, on which each patient case is reported
cSt: Centistoke, the unit of viscosity
D: Day, days of the week on which an activity is recorded
G6PD: Glucose 6-phosphate dehydrogenase deficiency, a genetic disorder
h: Hour, 60 min of daily time
IRCT: Iranian registry of clinical trials, a national website for clinical trial registry
ITT: Intention to treat, the population aimed to be treated
kdr: Knock-down resistance, a gene involved in resistance to an insecticide
km: Kilometer, unit of distance equal to 1000 m
m: Meter, standard unit of distance equal to 100 cm
min: Minute, one sixtieth of an hour
ml: Milliliter, a thousandth of a liter
OTC: Over the counter, a product delivered at a drug store
*P*: Probability, a measure of statistical likelihood
PDC: Plastic detection comb, a special hair comb for lice detection
PP: Per protocol, the population who completed a treatment protocol
SPSS: Statistical package for social sciences, a package for data analyses
